# Systemic antibody responses against human microbiota flagellins are overrepresented in chronic fatigue syndrome patients

**DOI:** 10.1126/sciadv.abq2422

**Published:** 2022-09-23

**Authors:** Thomas Vogl, Iris N. Kalka, Shelley Klompus, Sigal Leviatan, Adina Weinberger, Eran Segal

**Affiliations:** ^1^ Department of Computer Science and Applied Mathematics, Weizmann Institute of Science, Rehovot, Israel.; ^2^ Department of Molecular Cell Biology, Weizmann Institute of Science, Rehovot, Israel.; ^3^ Diagnostic and Research Institute of Hygiene, Microbiology and Environmental Medicine, Medical University Graz, Graz, Austria.

## Abstract

Myalgic encephalomyelitis/chronic fatigue syndrome (ME/CFS) is a debilitating disease with an unclear etiology and pathogenesis. Both an involvement of the immune system and gut microbiota dysbiosis have been implicated in its pathophysiology. However, potential interactions between adaptive immune responses and the microbiota in ME/CFS have been incompletely characterized. Here, we profiled antibody responses of patients with severe ME/CFS and healthy controls against microbiota and viral antigens represented as a phage-displayed 244,000 variant library. Patients with severe ME/CFS exhibited distinct serum antibody epitope repertoires against flagellins of *Lachnospiraceae* bacteria. Training machine learning algorithms on this antibody-binding data demonstrated that immune responses against gut microbiota represent a unique layer of information beyond standard blood tests, providing improved molecular diagnostics for ME/CFS. Together, our results point toward an involvement of the microbiota-immune axis in ME/CFS and lay the foundation for comparative studies with inflammatory bowel diseases and illnesses characterized by long-term fatigue symptoms, including post–COVID-19 syndrome.

## Teaser

Serum antibody responses against gut bacteria in patients with severe ME/CFS point toward an involvement of the microbiota-immune axis.

## INTRODUCTION

Patients with myalgic encephalomyelitis/chronic fatigue syndrome (ME/CFS) experience a lingering fatigue that can drastically impair their social and work life (*1*). ME/CFS affects approximately 1% of the population (*2*), appearing as a heterogeneous disease, with its etiology and pathogenesis still remaining elusive. Several biological processes ranging from energy production [e.g., metabolism (*3*) and mitochondrial dysfunction (*4*)] to neuroendocrinological aspects (*5*–*7*) have been implicated in ME/CFS. In addition, a potential involvement of the gut microbiota, enteric dysbiosis, increased gut permeability, and bacterial translocation has been suggested by several studies [e.g., (*8*–*11*)].

Given the diverse set of symptoms experienced by patients with severe ME/CFS beyond a debilitating fatigue [including sleep problems, muscle/joint pains, sore throat, and digestive issues such as irritable bowel syndrome (*12*)], diagnosis is typically achieved by questionnaires rather than molecular markers (*13*). Therefore, different efforts have aimed at identifying biomarkers for CFS, including immune markers (*14*) such as blood cytokine levels (*15*, *16*) as well as metabolic features [with some conclusions being controversially discussed (*17*–*21*)].

Several of these biomarkers, as well as similarities of ME/CFS symptoms to infections with pathogens (*22*), point toward an involvement of the immune system and inflammation in the pathophysiology of ME/CFS (*23*–*29*). It has been suggested that infections by viruses or bacteria can lead to immune dysregulation, manifesting in the observed fatigue symptoms (*13*). Epstein-Barr virus infection has been associated with ME/CFS symptoms since the 1980s (*30*–*33*). Recently, SARS-CoV-2 (severe acute respiratory syndrome coronavirus 2) has also been reported to cause persisting ME/CFS–like symptoms in a subset of recovered patients [termed as long coronavirus disease 2019 (long COVID), post–COVID-19 syndrome, or chronic COVID syndrome] (*34*–*38*), with implications for studying “classical” ME/CFS (*39*). Increased translocation of intestinal bacterial species into systemic translocation (*40*) and elevated systemic antibody responses against bacterial lipopolysaccharide (LPS) in ME/CFS (*41*) could also point toward immune responses against gut microbiota as a potential trigger for disease development (*10*). Beyond microbes, a potential involvement of autoimmunity has also been discussed, as ME/CFS is associated with hypothyroidism and Sjögren’s syndrome (*42*, *43*).

From a mechanistic perspective, recent works have highlighted antibody-producing B cells as a potential key branch of the adaptive immune system involved in the pathogenesis of ME/CFS (*44*, *45*). Sato *et al.* (*44*) sequenced the B cell receptor (BCR) genes of patients with severe ME/CFS and noticed pronounced differences compared to healthy controls. Milivojevic *et al.* (*45*) leveraged an orthogonal plasma proteomic approach [ultra-performance liquid chromatography–tandem mass spectrometry (UPLC-MS/MS)] pointing also toward an association between ME/CFS and the use of certain immunoglobulin (Ig) heavy variable genes. However, these BCR sequencing (BCR-seq) and UPLC-MS/MS approaches only report on the clonality and diversity of BCRs or fragments of serum antibodies, while the actual antigens recognized remain enigmatic.

Here, we have applied a high-throughput functional antibody assay [phage immunoprecipitation sequencing (PhIP-Seq) (*46*)] to compare the Ig epitope repertoires of patients with severe ME/CFS and healthy controls against a library of 244,000 bacterial and viral epitopes (*47*). Patients with severe ME/CFS exhibited distinct serum antibody responses against flagellins of *Lachnospiraceae* bacteria, which could be leveraged alongside machine learning algorithms as potent diagnostic markers.

## RESULTS

### PhIP-Seq assay and cohorts of patients with severe ME/CFS and healthy controls

Conventional serological methods such as enzyme-linked immunosorbent assays (ELISAs) or peptide arrays allow analysis of hundreds or thousands of antigens in parallel (*48*), representing suitable strategies to test antibody responses against larger sets of candidate antigens of interest. However, in ME/CFS, a wide range of viral and bacterial antigens (including species of the gut microbiota) have been speculated to be involved (*22*–*29*), with the gut microbiota especially representing a vast space of potential antigens [thousands of species, with each species encoding thousands of genes (*49*)].

Therefore, we leveraged a high-throughput antibody profiling technology, PhIP-Seq (*46*), to test for reactivity against 244,000 peptide antigens (*47*) in a target-agnostic way (*50*). This antigen library encompasses diverse bacterial and viral antigens originating from pathogenic, probiotic, and commensal bacteria, including antigens selected from metagenomic sequencing [see (*47*) and Methods for details]. The library also includes all B cell antigens of pathogens from the Immune Epitope Database [IEDB, the largest resource for previously reported antigens (*51*)] as well as bacterial virulence factors from the Virulence Factor Database [VFDB (*52*)].

**Fig. 1.**
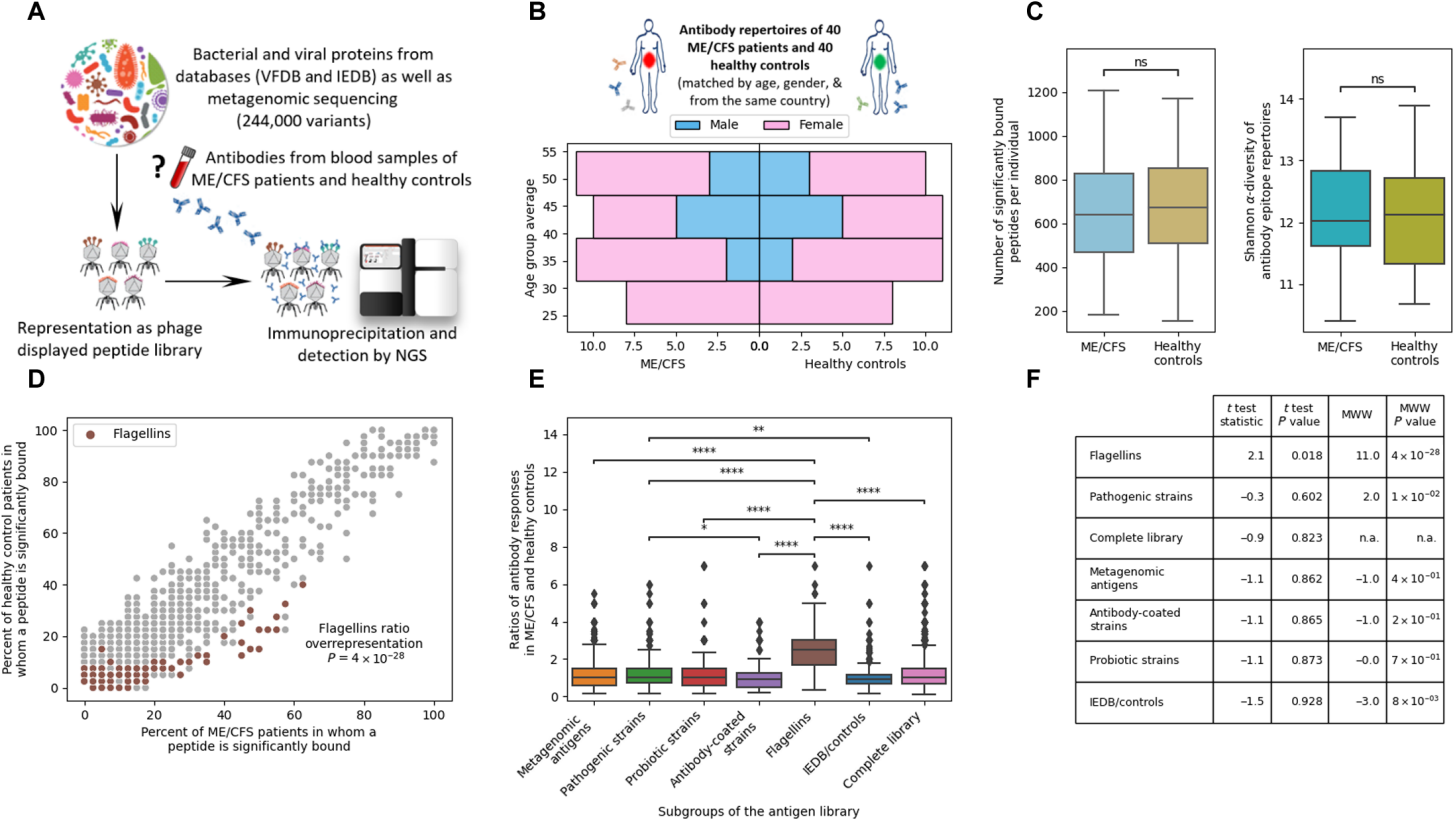
Analysis of antibody responses against 244,000 bacterial and viral peptide antigens indicates aberrant Igs against flagellins as the key difference between patients with severe ME/CFS and healthy controls. (**A**) PhIP-Seq (*46*) methodology to analyze serum antibody epitope repertoires against a diverse library (*47*) of peptide antigens. (**B**) Blood samples for antibody profiling were obtained from 40 patients with severe ME/CFS and an equal number of age- and sex-matched healthy controls. (**C**) The absolute number of antibody-bound peptides per patient and the diversity of Ig repertoires were not significantly different between patients with severe ME/CFS and healthy controls. See Methods for details on the statistics applied. (**D**) Antibody responses against bacterial flagellins are significantly [*t*_(78)_ = 11.0, *P* = 4 × 10^−28^] overrepresented in patients with severe ME/CFS compared to healthy controls. Each dot represents a peptide, with its prevalence in the respective cohort plotted on the *x* and *y* axes. Bacterial flagellins as previously annotated (*47*) are marked. See table S1 for a detailed list. When applying Fisher’s exact test to test for the differences between patients with severe ME/CFS and healthy controls, no peptides were significantly enriched (after false discovery rate for the 20,694 peptides bound in at least one person of the cohort). (**E** and **F**) Bacterial flagellins represent the main antigen subgroup within the library that exhibits differential binding in patients with severe ME/CFS and healthy controls (see Methods for details). ns, not significant. *P ≤ 0.05, **P ≤ 0.01, ***P ≤ 0.001, ****P ≤ 1.00 × 10^−04^. n.a., not applicable.

Methodologically, PhIP-Seq is based on the parallelized detection of antibody responses against phage-displayed antigens by immunoprecipitation and a next-generation sequencing–based readout (Fig. 1A). Applications of this technology have provided unprecedented insights into antiviral (*53*–*56*) and antibacterial immune responses (*57*), including detection of a breadth of serum antibody responses against gut microbiota (*47*).

We applied a PhIP-Seq workflow to compare the Ig epitope repertoires of 40 patients with severe ME/CFS and an equal number of matched healthy controls (Fig. 1B). Given the variability and uncertainty in diagnosis of ME/CFS, our cohort was represented by severe ME/CFS cases uniformly assembled by the U.K. ME/CFS Biobank (UKMEB) (*58*). As antibody epitope repertoires are affected by age and sex (*47*), the 40 healthy controls were 1:1 matched to the ME/CFS cases to eliminate any bias (Fig. 1B). The healthy controls were recruited by the UKMEB, reducing potential biases related to geography or sample handling.

### Anti-flagellin Ig responses are overrepresented in patients with severe ME/CFS

Comparing general metrics such as the number of overall antibody-bound peptides or the diversity of the Ig epitope repertoires did not show any significant differences between patients with severe ME/CFS and healthy controls (Fig. 1C). Mann-Whitney-Wilcoxon tests indicated that the difference in both number of significantly bound peptides per individual and Shannon α-diversity were not statistically significant (*P* > 0.3 and *P* > 0.9, respectively). In addition, no single peptides were bound at significantly different rates in patients with severe ME/CFS and healthy controls after false discovery rate correction (Fig. 1D, Fisher’s exact test for differences between the two groups). However, the majority of peptides, which were more frequently bound in patients with severe ME/CFS, originated from bacterial flagellins (Fig. 1D and table S1 for a full list).

To test whether these differences for antibody binding against flagellins are significantly different, we performed multiple statistical tests (Fig. 1, D to F). These tests were also extended beyond flagellins to other subgroups of the antigen library to test, for example, whether pathogenic bacteria, metagenomic antigens, etc. are also overrepresented in patients with severe ME/CFS. First, by applying a *t* test on the ratio of patients with severe ME/CFS versus healthy controls for binding each peptide showed that the group of flagellins was significantly overrepresented in patients with severe ME/CFS compared to healthy controls in comparison to all other peptides in the complete library (Fig. 1D, *P* < 4 × 10^−28^). Next, we compared the healthy controls and the patients with severe ME/CFS by performing a rank sum test on the number of peptides that were signifyingly bound in each individual to evaluate whether other groups of antigens also showed differences, confirming the overrepresentation of flagellins by an alternative statistical approach (Fig. 1E). Overall, antibody responses against flagellins were much more significantly overrepresented in patients with severe ME/CFS versus healthy controls than any other antigen group (*t* test results summarized in Fig. 1F).

To summarize the results of all statistical tests, we found that antibody responses against the group of flagellins are significantly overrepresented in patients with severe ME/CFS versus healthy controls when compared to all other peptides (Fig. 1D). The same overrepresentation of flagellins is evident when comparing pairs of antigen groups (Fig. 1E). Last, in Fig. 1F, results of rank sum (comparison of antibody responses of patients with severe ME/CFS and healthy controls for a specific group of antigens) are summarized.

**Fig. 2.**
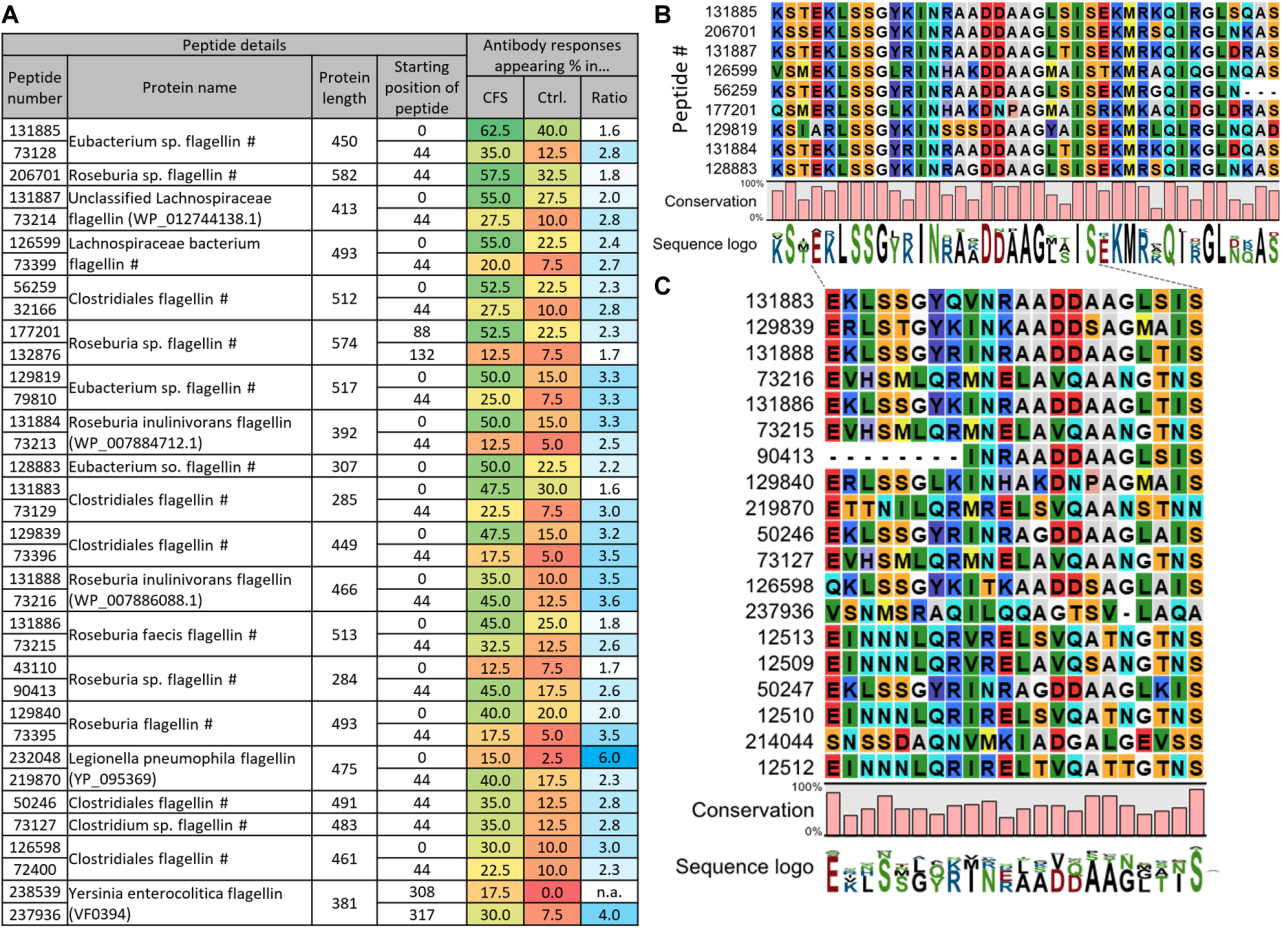
Bacterial flagellins bound by antibodies disproportionally in patients with severe ME/CFS stem from the family of *Lachnospiraceae* and share an N-terminal motif. (**A**) Bacterial flagellins that exhibited antibody responses in ≥30% of CFS patients. Additional peptides per protein are shown if they appeared in >4 individuals. Peptide number: #, Uniref-derived annotation (see Methods). Ratio: Ratio of prevalence in CFS/healthy (Ctrl., control). See table S1 for a full list of all flagellin peptides. (**B** and **C**) Alignments of bound flagellin peptides suggest shared motifs bound by cross-reactive Ig responses. In (B), alignments of peptides appearing in ≥50% of CFS patients are shown. In (C), peptides appearing in ≥25% are shown. The peptides from (B) were also used in (C) but are not shown due to space constraints. See fig. S1 for full alignments. Alignments were generated with MegaX [MUSCLE algorithm in standard settings (*81*)].

Next, we studied the phylogenetic origin of the antibody-bound flagellins in greater detail. The overrepresented flagellins were frequently originating from the order of *Clostridiales* and mostly the family of *Lachnospiraceae* (species such as *Roseburia inulinivornas* or *Roseburia faecis*) as well as some *Eubacteriaceae* (genus *Eubacterium*). In addition, some flagellins of *Gammaproteobacteria* (e.g., genera of *Salmonella*, *Pseudomonas*, and *Escherichia*) were more frequently bound by antibodies in patients with severe ME/CFS. A summary of the most frequently bound flagellins is shown in Fig. 2A, while a full list is provided in table S1. On average, antibody responses against these flagellins were 2.2-fold more frequent in patients with severe ME/CFS than in healthy controls (table S1). Some variants such as a *Roseburia inulinivorans* flagellin (peptide no. 129819) elicited antibody responses in 50% of patients with severe ME/CFS but only 15% of healthy controls (3.3-fold overrepresentation). Frequently, two overlapping N-terminal peptides of the same protein were bound (such as peptide pairs #131885 and #73128, #131887 and #73214, or #126599 and #73399 shown in Fig. 2A).

Such overlaps can exist since, when designing our antigen library, we tiled larger proteins into adjacent peptides of 64 amino acids with overlaps of 20 amino acids (*47*). Hence, the observed antibody binding likely occurs against an epitope in the overlaps. Alignments of the peptides indeed showed a shared motif (Fig. 2, B and C, and fig. S1; by applying two different cutoffs), potentially recognized by cross-reactive antibodies. We had previously observed cross-reactivity of human monoclonal antibodies against viral antigens with a different PhIP-Seq library (*59*). Hence, the observed overrepresentation of Ig responses against flagellins in patients with severe ME/CFS is not necessarily triggered by exposure to all the observed bacterial species, but a subgroup or even a single species could suffice.

### Machine learning on Ig epitope repertoires diagnoses severe ME/CFS

Given these distinct differences in antibody responses of patients with severe ME/CFS and healthy controls, we aimed to leverage these Ig epitope repertoires for diagnostics. Identifying a representative set of differentially bound peptides, allowing separation of patients with severe ME/CFS from controls, could yield potent biomarkers. However, since our PhIP-Seq implementation detected thousands of enriched antibody-bound peptides, it would be challenging to manually select the ones with the greatest discriminatory potential. Therefore, we leveraged machine learning algorithms [gradient boosting regression (GBR) (*60*) and XGBoost (XGB) (*61*)] to select the optimal set of antibody responses yielding the highest diagnostic accuracy.

**Fig. 3.**
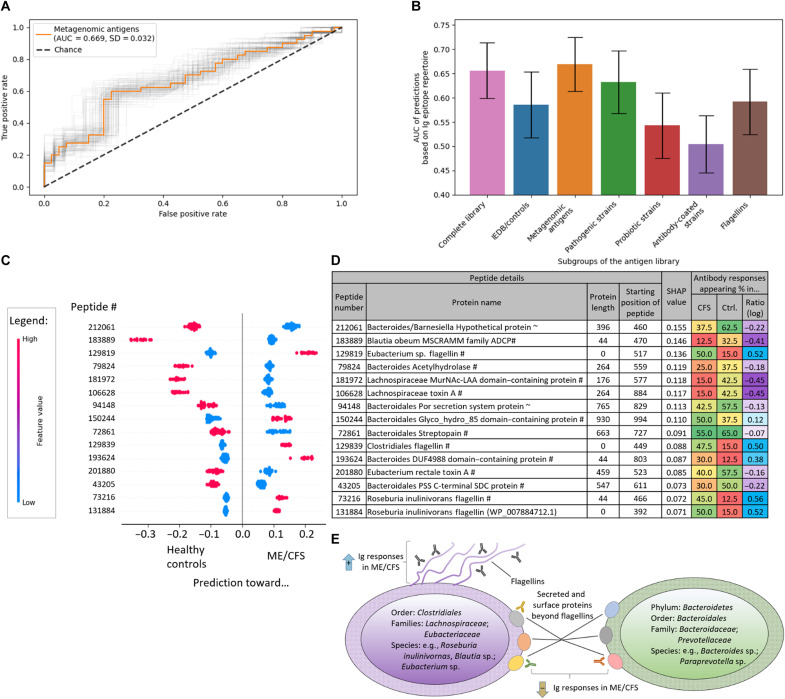
Machine learning algorithms trained on Ig epitope repertoires separate patients with severe ME/CFS from healthy controls. The classifications are driven by antibody responses against *Lachnospiraceae* flagellins overrepresented in patients with severe ME/CFS as well as antibody responses against other surface proteins of *Bacteroidetes* and *Lachnospiraceae* in the healthy controls. All predictions were performed with GBR (*60*) and leave-one-out cross-validation on different prevalence cutoffs of antibody responses appearing in the cohorts (see Methods for details). (**A**) Receiver operator curve (ROC) of the best-performing combination of antibody-bound peptides and prevalence cutoffs. The light gray lines illustrate ROC confidence intervals (CIs, 95%) as computed by bootstrapping. (**B**) Summary of model performance trained on antibody responses against different subsets within the antigen library. See Methods for details on the subgroups of the antigen library. Error bars represent 95% CIs as in (A). (**C** and **D**) SHAP analysis of the best-performing model [shown in (A)]. The top 15 contributing peptides are shown (C), and details are provided (D). See table S3 for a full list. Every line represents a peptide, and in every line, every dot represents the prediction result contribution for one individual (*n* = 80; 40 CFS patients and 40 controls). See Methods for abbreviations. (**E**) Schematic illustration of antibody responses against the respective proteins from *Lachnospiraceae*, *Eubacteriaceae*, and *Bacteroidetes* driving classification.

As we had observed that some groups of antigens such as flagellins and proteins annotated from metagenomic sequencing showed greater differences in Ig epitope repertoires (Fig. 1), we ran the machine learning algorithms on the entire set of antibody-bound peptides, as well as the subgroups (Fig. 3, A and B, and table S2). Models trained on the subgroup of metagenomics data [area under the received operator curve (AUC) = 0.67; Fig. 3A] slightly surpassed analyses on all antigens (AUC = 0.66) as well as from the subgroup of flagellins (AUC = 0.59). The superior performance of metagenomic antigens compared to the entire library may be due to a removal of irrelevant features that may only increase noise and not contribute to the separation of patients with severe ME/CFS from healthy controls. We performed additional validations to rule out any bias of age or sex difference (fig. S2) as well as potentially greater performance by other models (fig. S3).

Antigens selected from metagenomic sequencing appear to carry additional information beyond the flagellins that primarily appeared differentially bound when comparing raw Ig epitope repertoire differences (Figs. 1 and 2). To gain insights into additional antibody responses contributing to the separation between patients with severe ME/CFS and healthy controls, we performed a SHAP (SHapley Additive exPlanations) analysis (*62*) on the best-performing model (Fig. 3, C and D). While many machine learning algorithms on their own represent a “black box,” making it difficult to understand factors contributing to model output, SHAP analysis allows for disentanglement of the effect of single features on the prediction. As expected, several top-ranking peptides were flagellins from *Clostridiales* (Fig. 3C and table S4). Peptides from several surface proteins of bacteria from the phylum of *Bacteroidetes* (specifically the order of *Bacteroidales*) also ranked highly among contributing factors. While some of these proteins were poorly annotated, nearly all of them contained conserved domains associated with surface exposure or secretion such as peptide no. 94148 originating from a Por secretion system (PSS) protein, no. 150244 stemming from a protein containing a PSS C-terminal sorting domain, or several hydrolases for glycoside breakdown. Most of these proteins showed increased antibody binding in healthy individuals compared to patients with severe ME/CFS (Fig. 3D). A similar pattern was also evident for nonflagellin surface and secreted proteins of *Clostridiales* since also overrepresented in healthy controls to patients with severe ME/CFS were antibody responses against toxin A from *Lachnospiraceae* and *Eubacterium*, an adhesin protein from *Blautia obeum*, and a *Lachnospiraceae* MurNAc-LAA (*N*-acetylmuramoyl-l-alanine amidase) domain containing protein involved in cell wall modifications. In some cases, even multiple peptides from the same protein appeared in the SHAP analysis (e.g., peptide nos.106628 and 209928; table S3), making it highly unlikely that these Ig responses against very specific bacterial taxa and protein groups would occur by chance.

These results indicate that microbial proteins beyond flagellins and species beyond *Lachnospiraceae* contribute to the differential antibody recognition between patients with severe ME/CFS and healthy controls. Notably, antibody binding of flagellins follows an opposing pattern observed for *Bacteroidetes* proteins and nonflagellin surface proteins from *Lachnospiraceae* (summarized in Fig. 3E): While patients with severe ME/CFS exhibit increased antibody responses against the former, the latter are overrepresented in healthy individuals and lower in patients with severe ME/CFS. These data may point toward a regulatory role of antibody responses against different surface proteins in healthy individuals. Lack of these antibodies could be associated with a potential overreaction against flagellins in patients with severe ME/CFS.

### Combining conventional blood tests with Ig epitope repertoires improves diagnosis

**Fig. 4.**
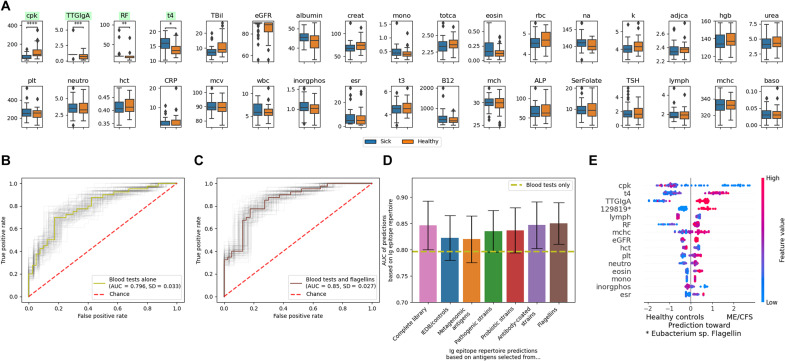
Antibody responses against gut microbiota represent a unique layer of information beyond conventional blood tests, allowing for improved diagnosis of ME/CFS by machine learning algorithms. (**A**) Blood test results of patients with severe ME/CFS and healthy controls. Significantly different (Mann-Whitney-Wilcoxon test with Bonferroni correction) tests are highlighted in green. Missing blood tests were removed from the comparisons. Mann-Whitney-Wilcoxon test two-sided results: *U*(N_control_ = 40, *N*_ME/CFS_ = 40) = 291; *P* < 1 × 10^−5^). *P ≤ 0.05, **P ≤ 0.01, ***P ≤ 0.001, ****P ≤ 1.00 × 10^−04^. See Methods for details and table S4 for abbreviations used and a full description of the respective blood test markers. (**B**) Machine learning algorithms on blood tests alone allow to classify patients with severe ME/CFS from healthy controls. ROC with the light gray lines illustrating model variability by bootstrapping (as in Fig. 3A). (**C** and **D**) Addition of antibody repertoire data improves diagnosis compared to conventional blood tests alone. In (C), an ROC of the best-performing combination of antibody bound peptides and prevalence cutoffs is shown [error bars represent 95% CIs as in (B)]. (D) Summary of model performance trained on antibody responses against different subsets within the antigen library. See Methods for details on the subgroups of the antigen library. (**E**) SHAP analysis of the best-performing model [shown in (C)]. The top 15 contributing features are listed. See table S6 for a full list, and see Fig. 3C for an explanation of the SHAP analysis. Predictions in (C) to (E) were performed with XGB (*61*) and leave-one-out cross-validation on different prevalence cutoffs of antibody responses appearing in the cohorts (see Methods for details).

While antibacterial antibody responses in patients with severe ME/CFS showed promise to gain etiological and pathophysiological insights (Fig. 3), we aimed to assess their potential diagnostic potential in comparison with previously reported molecular markers. Conventional blood tests covering routinely assessed hematological and biochemical parameters show substantial differences between patients with severe ME/CFS and healthy controls (*63*). As a variety of blood tests had been run on our cohorts from the UKMEB, we compared these results between patients with severe ME/CFS and healthy controls (Fig. 4A). The most notable difference was in creatine kinase concentration, which was significantly (two-sided Mann-Whitney-Wilcoxon, *P* < 1 × 10^−5^) lower in patients with severe ME/CFS than in healthy controls, as previously reported for samples from the UKMEB (*63*).

Next, we trained machine learning algorithms (XGB and GBR) to separate between cases and controls solely based on blood test results as features, yielding an AUC of 0.80 (Fig. 4B). This result surpassed the best-performing prediction from antibody repertoires (AUC = 0.67; Fig. 3A). However, combining conventional blood test results with antibody repertoire data significantly (outside 95% confidence interval computed by bootstrapping) improved predictions based on blood tests alone (Fig. 4, C and D). Next, we combined the blood test data with different subgroups of the antigen library (Fig. 4D), revealing bacterial flagellins to yield the highest AUC of 0.85 (Fig. 4C). A SHAP analysis also identified antibody responses against a flagellin from *Eubacterium* (peptide no. 129819) as a key feature to this outcome (Fig. 4E and table S6). The GBR algorithm (*60*) performed worse than XGB (*61*), possibly as XGB is a more advanced version of GBR allowing to deal with missing values without the need to perform imputation (the blood tests, which were added for these predictions, involved missing values for several samples; fig. S4 and table S5).

While XGB predictions based on blood tests alone (AUC = 0.80) surpassed antibody repertoire data alone (AUC = 0.67), their combination yielded synergistic effects (AUC = 0.85). Antibody responses against flagellins allowed to classify 25% in addition to blood tests alone. Hence, antibody responses against gut microbiota detectable by PhIP-Seq represent a unique layer of information beyond standard blood tests, allowing for improved diagnosis of ME/CFS.

## DISCUSSION

### Antibody responses in ME/CFS versus CD

In this study, we have detected an overrepresentation of systemic anti-flagellin Ig responses in patients with severe ME/CFS by leveraging a target-agnostic (*50*) PhIP-Seq screening approach (*47*). A similar overrepresentation of serum Ig responses against *Lachnospiraceae* flagellins has been reported in Crohn’s disease (CD) (*64*, *65*). CD is a chronic inflammatory disease of the intestinal tract associated with intestinal microbial dysbiosis and immune system dysregulation (*66*). *Lachnospiracea*e and other *Clostridiales* produce short-chain fatty acids that mediate a range of beneficial effects in the gut such as enhancing tolerance and the epithelial barrier function, anti-inflammatory effects, as well as activating regulatory T cells (*64*, *67*). Hence, excessive, mistargeted immune reactions against these favorable commensals are conceptually in line with CD and irritable bowel syndrome, a common comorbidity reported by patients with severe ME/CFS (*10*). Notably, CD patients (compared to healthy individuals) have an increased risk to also develop ME/CFS (*68*).

However, while there are some symptomatic similarities between CD and ME/CFS, there are also key differences. The majority of CD patients do not experience the lingering fatigue and postexertional malaise characteristic of ME/CFS [although a subset of CD patients do report fatigue (*69*)]. Vice versa, patients with severe ME/CFS typically do not experience severe gastrointestinal symptoms occurring in CD [while IBS (irritable bowel syndrome) is commonly reported (*10*)]. It appears puzzling that both diseases would share an involvement of similar antimicrobial immune responses and display different phenotypes, pointing toward additional genetic or environmental factors involved. However, we also did notice apparently unique antibody responses in patients with severe ME/CFS, which have, to the best of our knowledge, not been reported in CD patients. Among these, systemic antibody responses against *Bacteroidetes* surface proteins tended to be less frequent in patients with severe ME/CFS than in healthy controls. Notably, Ig responses against nonflagellin surface- and secreted-proteins from *Clostridiales* were also underrepresented in patients with severe ME/CFS. We had analyzed CD patients’ Ig repertoires against the same PhIP-Seq library, and while we did detect similar flagellins, differential antibody responses against other *Clostridiales* and *Bacteroidetes* surface proteins were not apparent (*65*), making varying experimental workflows (*64*) or biased antigen selection unlikely accountable for the differences observed.

This antibody-binding pattern may point toward an antigen-specific overreaction in ME/CFS: While in healthy individuals, both flagellins and other proteins from *Lachnospiraceae* are Ig bound at moderate rates, flagellins dominate the Ig epitope repertoires of patients with severe ME/CFS. Beyond *Lachnospiraceae*, the lower antibody responses against *Bacteroidetes* in patients with severe ME/CFS appear counterintuitive on first sight, as *Bacteroidetes* are generally associated with beneficial effects on human health. More frequent antibody responses in healthy individuals compared to patients with severe ME/CFS potentially clearing these species would appear teleologically unfavorable. However, we have previously also detected rather frequent systemic Ig responses against various *Bacteroidetes* in healthy individuals, with these antibody responses being potentially indicative of the presence of these bacteria and not necessarily associated with harmful effects (*47*). In this line of thought, *Bacteroidetes* abundances have been reported to be decreased in metagenomic sequencing in a different cohort of patients with severe ME/CFS (*10*, *70*), potentially supporting this notion. Elevated *Lachnospiraceae* abundances have not explicitly been reported in gut microbiome sequencing of patients with severe ME/CFS [see (*10*, *71*) for comprehensive reviews of gut microbiota changes in ME/CFS], pointing toward a peculiar role of anti-*Lachnospiraceae* Ig responses. However, stool samples or metagenomic data are unavailable for the individuals whose blood samples were used in our study. So, it is not directly possible to investigate such links for this cohort.

### Limitations of the study

The Ig epitope repertoire profiling efforts outlined here for ME/CFS are constrained by technical limitations of PhIP-Seq previously discussed in detail [e.g., (*46*, *47*)]. Our PhIP-Seq library relies on presentation of 64 amino acid peptides. Hence, larger conformational epitopes, structures not folding correctly within 64 amino acid segments, nonprotein antigens (such as glycans or lipids), or posttranslational modifications would be missed. However, linear epitopes are expected to be well represented, and ultimately, the ratio of linear to conformational epitopes is difficult to assess. Even if our PhIP-Seq implementation were only capable of detecting 10% of antibody-antigen interactions in ME/CFS, this approach would still exceed conventional efforts based on ELISAs or peptide arrays by an order of magnitude. Antibody binding against single peptides detected by PhIP-Seq needs to be carefully evaluated and verified with orthogonal methods (*47*). Key binding events reported in this study as responses against flagellin (Fig. 2) and other surface structures (Fig. 3D) were corroborated by binding of multiple, in part overlapping peptides per protein and several functionally related proteins from phylogenetically closely related species, making it highly unlikely that these signals would co-occur by chance.

Overall, our cohort of patients with severe ME/CFS consisted of severe cases only, and Ig epitope repertoires showed substantial variability, with classifications based on antibody repertoires yielding an AUC of 0.67 (Fig. 3A)—far from a perfect separation. Assaying a larger cohort could potentially improve the predictions (*63*). Also, addition of data from conventional blood tests as features left some uncertainty (AUC = 0.85; Fig. 4C). This result may point toward subgroups of disease phenotypes existing within the umbrella term of ME/CFS, possibly caused by yet unknown genetic or environmental factors. However, our data suggest at least the existence of a ME/CFS patient subpopulation with an imbalance of systemic antimicrobiota-directed Ig responses. Such heterogeneity is also known from other immune-mediated diseases. For example, antibody responses in CD were also reported to be variable with only a subset of patients showing high responses against several flagellins tested (*64*). Hence, leveraging larger ME/CFS cohorts, including different grades of disease severity as well as multiomic analyses, could identify disease subgroups more clearly and may help to stratify patient populations for treatments.

Furthermore, our data do not inform on a causal role of the antibody responses detected in the etiology of ME/CFS. The observed increased antibody binding against flagellins could be a collateral side effect downstream of disease onset. For example, differences in the conventional blood test marker creatine kinase (a marker for muscle activity significantly decreased in patients with severe ME/CFS (*63*); Fig. 4A) have been speculated to be caused by physical inactivity of patients with severe ME/CFS (*63*). Likewise, antibody responses against microbiota may be affected by other factors: Dietary changes due to loss of appetite could affect the gut microbiota composition or extensive resting periods, and impaired mobility could lead to increased intestinal permeability and bacterial translocation eliciting systemic antibody responses. However, even if arising from downstream effects, these Ig responses nonetheless represent powerful molecular markers improving diagnosis of severe ME/CFS cases beyond conventional approaches.

### Diagnostic potential and outlook

Despite all the limitations outlined in the last section, our data provide a link between an involvement of gut microbiota (*10*) and aberrant antibody repertoires detected by BCR-seq (*44*, *45*) in ME/CFS. Both an involvement of gut microbiota and of the adaptive immune system have been implicated in the pathogenesis of ME/CFS. Enteric dysbiosis, increased gut permeability, and bacterial translocation have been reported in patients with severe ME/CFS compared to healthy individuals [reviewed in (*10*)]. In addition, increased serum IgA and IgM concentrations against bacterial LPS have been detected in patients with severe ME/CFS, and BCR-seq studies, representing the genetic basis for antibody binding, have indicated differences in patients with severe ME/CFS compared to healthy individuals (*44*, *45*). However, the actual bacterial or viral antigens targeted by Ig responses in patients with severe ME/CFS had been incompletely characterized.

Therefore, our work demonstrates that PhIP-Seq can be used as a powerful tool to mine for immune biomarkers in a target-agnostic way (*50*), as we leveraged a broad antigen library created from databases and metagenomic sequencing of healthy individuals (*47*) without any specific preconceptions of ME/CFS. Despite the smaller cohort size compared to other efforts (*47*, *59*, *65*), the PhIP-Seq workflow yielded sufficiently sensitive detection thresholds and accuracy to generate previously unidentified insights into the relatively little researched disease ME/CFS. Training machine learning algorithms on this Ig epitope repertoire data showed that immune responses against gut microbiota represent a unique layer of information beyond standard blood tests, allowing for improved molecular diagnosis of ME/CFS.

These findings can be interpreted toward disease mechanisms, as Ig responses in patients with severe ME/CFS against *Lachnospiraceae* were dominated by binding against flagellins, while Ig respones against other surface proteins of this family and *Bacteroidetes* were underrepresented compared to healthy controls. These observations highlight both similarities and differences to IBD, where only an overrepresentation of anti-flagellin responses has been reported (*64*, *65*). This finding may indicate a different mechanism in ME/CFS, where lack of neutralization of other surface targets may trigger an overreaction against flagellins. Supporting this notion, varying effects elicited by antibody binding of different bacterial surface proteins have been shown in animal models (*72*).

While the focus of this work lies in diagnostics, these observations point also toward potential therapeutic strategies since the identified ME/CFS–specific antibody responses could be leveraged for prognosis and targeted in future personalized therapies [as discussed similarly for CD (*64*)]. Such efforts may include preventive vaccination with *Lachnospiraceae* or *Bacteroidetes* surface antigens to counteract anti-flagellin overreaction as well as therapies reducing anti-flagellin binding.

Ultimately, findings from this study may also be relevant to gain insights into long COVID, where a subset of patients who recovered from SARS-CoV-2 infection experience persisting ME/CFS–like fatigue symptoms (*34*–*39*). Genetic or environmental factors affecting the development of long COVID are vastly unknown, with PhIP-Seq screening for Ig responses against microbiota (*47*) antigens observed in classical ME/CFS as well as coronaviruses (*55*, *59*) representing a potential avenue to pursue.

## METHODS

### Serum samples and blood tests

Serum samples of 40 severe ME/CFS cases and 40 healthy controls were obtained from the UKMEB (*58*). As antibody epitope repertoires are affected by age and sex (*47*), the healthy controls were 1:1 matched to the ME/CFS cases to eliminate any bias. The healthy controls had also been recruited by the UKMEB, reducing potential biases related to geography or sample handling (identity of matched pairs was not specified). Research with these samples has been approved by the Weizmann Institute of Science’s institutional review board (#1410-2), and the donors had consented to research use of the samples.

A variety of conventional blood tests had also been obtained for our cohorts by the UKMEB (*58*, *63*), and we used these data in this study. Details on the exact hematological and biochemical parameters assessed (as well as ranges) are provided in table S4. Tests, where more than 10 individuals had missing data, were removed from the analysis (marked in table S4).

### Content of the PhIP-Seq microbiota antigen library, immunoprecipitation, and sequencing

We used a 244,000-variant phage display library previously created with design considerations, and the exact antigens included were detailed in (*47*). In short, this antigen library encompasses diverse bacterial and viral antigens originating from common gut pathogens (*47*, *73*), probiotics [strains from a recent review (*74*)], gut microbiota previously reported to be coated by antibodies (*75*), and commensal bacteria, including antigens selected from metagenomic sequencing of nearly 1000 healthy individuals (*76*). We have also included all B cell antigens of pathogens from the IEDB [the largest resource for previously reported antigens (*51*)] as well as bacterial virulence factors from the VFDB (*52*).

Proteins from these species were functionally prioritized, including the groups of membrane proteins, secreted proteins, and motility proteins/flagella [the detailed process is described in (*47*)]. The flagellins studied in this work are based on this annotation. There are a few additional flagellins (originating from databases) that were not grouped in this way and manually added to table S1 (see column there). Especially for genes obtained from metagenomic sequencing, in part no functional annotations were available. Hence, protein functions were annotated by mapping to the UniRef90 database (uniref and uniref_func) (*47*), which is marked if applicable in figures and tables.

The PhIP-Seq experiments were carried out as outlined in a published protocol (*46*) with laboratory-specific modifications detailed in (*47*). In short, in each reaction, 3 μg of IgG from a person’s serum sample (concentration measured by ELISA) was added to the phage library (4000-fold coverage of phages per library variant). The microbiota library was mixed in a 2:1 ratio with a 200-nucleotide oligomer 100,000 variant pool (*65*, *77*).

The antibody-phage mixture was mixed at 4°C overnight on a rotator. Forty microliters of a 1:1 mixture of protein A and G magnetic beads (Thermo Fisher Scientific, catalog nos. 10008D/10009D, washed according to the manufacturer’s recommendations) was added after overnight incubation and incubated on a rotator at 4°C. All 80 samples were processed in the same 96-well plate. After 4 hours, the beads were transferred to polymerase chain reaction (PCR) plates and washed twice as previously reported (*46*) using a Tecan Freedom Evo liquid handling robot with filter tips. PCR amplifications for pooled Illumina amplicon sequencing were performed with Q5 polymerase (New England Biolabs, catalog no. M0493L) according to the manufacturer’s recommendations [primer pairs PCR1: tcgtcggcagcgtcagatgtgtataagagacagGTTACTCGAGTGCGGCCGCAAGC and gtctcgtgggctcggagatgtgtataagagacagATGCTCGGGGATCCGAATTC; PCR2: Illumina Nextera combinatorial dual index primers, PCR3 (of PCR2 pools): AATGATACGGCGACCACCGA and CAAGCAGAAGACGGCATACGA (*46*); custom sequencing primers for R1: ttactcgagtgcggccgcaagctttca and for R2: tgtgtataagagacagatgctcggggatccgaattct, R1/R2 44/31 nts]. Paired end reads were processed, and statistical analysis was performed as previously outlined (*47*).

While it is possible to resolve binding profiles of different antibody classes with PhIP-Seq (*55*), our standard workflow based on protein A/G beads detects primarily IgG (*47*). Studying different antibody classes such as IgA not only in blood but also at mucosal sites may improve diagnostic power in ME/CFS as well as generate hypotheses on disease mechanisms.

### Machine learning and data analysis

We filtered the 244,000 peptides making up the entire antigen library to those significantly bound in at least one individual from the cohort, resulting in a total of 20,694 epitopes. We processed the PhIP-Seq data in two ways. The first is the fold change as defined in (*47*) (comparing the read number after antibody binding to the input library, a proxy for binding strength). The second is binary existence of binding (e.g., any fold change greater than 1).

We compared the distribution of antibody bound peptides between healthy controls and patients with severe ME/CFS by the number of epitopes each individual displayed and the Shannon-α diversity (*78*). We compared the two groups using Mann-Whitney-Wilcoxon tests.

For each subgroup of the antigen library, we performed multiple statistical tests. First, we compared the healthy controls and the patients with severe ME/CFS by performing a rank-sum test on the number of peptides that were significantly bound in each individual. This methodology is not biased by the number of antigens within the specific antigen subgroup. The second test we performed was a *t* test on the ratio between healthy controls and patients with severe ME/CFS. For this test, we filtered only for peptides that appeared in at least one healthy control and at least one ME/CFS patient. We then computed the ratio for each peptide. The distribution of ratios was compared using a *t* test between each subgroup of the antigen library and all other peptides. We also performed a Mann-Whitney-Wilcoxon comparisons between the ratios of each pair of subgroups (including the complete library).

We identified that one blood test (astbloodb) appeared in all but one patients with severe ME/CFS and in less than half of the healthy controls and removed it from our analyses. Blood test results were compared between healthy controls and patients with severe ME/CFS using a Mann-Whitney-Wilcoxon test and corrected for multiple testing using the Bonferroni method.

We performed classification of outcomes using both Scikit-learn (*79*) Gradient Boosting Classifier and XGB (*61*) classifier. When predicting with XGB, we used the following parameters: use_label_encoder = False, objective = “binary:logistic,” and eval_metric = “logloss.” In the case of GBR, we used the following parameters: n_estimators = 2000, learning_rate = 0.01, max_depth = 6, max_features = 1, and min_samples_leaf = 10. All other parameters remained default. Classifications were performed using a leave-one-out cross validation. For the first classifier, we performed imputation using Scikit-learn SimpleImputer to eliminate missing values. Predictions were performed multiple times filtering for peptides that appear in at least a threshold percent of individuals (thresholds used were 0, 1, 5, 10, 20, 50, 95, and 100%). Predictions were also performed on both fold change data and existence data. In our figures, we display the best result for each subgroup of the antigen library. The complete results appear in supporting tables (tables S2 and S5).

Performance of our models was evaluated using AUC. We computed the SD (and thus the 95% confidence intervals) of the AUC using bootstrapping of the predictions.

To interpret the models and the effect of features on predictions, we used SHAP TreeExplainer (*80*). From it, we obtained the Shapley additive explanation values of each feature, allowing us to identify the importance of the different features to the model. This analysis was performed using the best performance model parameters trained on the entire cohort.

### Statistics and machine learning details in Figs. 1, 2, and 4

In Fig. 1C, the center line of the boxplots shows the median. Box limits indicate the 25th and 75th percentiles. Whiskers extend to 1.5 times the interquartile range from the 25th and 75th percentiles. *n* = 40 for each group. Mann-Whitney-Wilcoxon two-sided tests were found to be not significant (*P* > 0.05) when comparing ME/CFS to control groups in both measurements. Number of significantly bound peptides per individual *U*(*N*_control_ = 40, *N*_ME/CFS_ = 40) = 704, *P* > 0.3; Shannon α-diversity *U*(*N*_control_ = 40, *N*_ME/CFS_ = 40) = 809, *P* > 0.9.

In Fig. 1E, the ratio (of the prevalence) between antibody bound peptides that appear in both at least one healthy control and at least one ME/CFS patient is presented. The *y* axis shows the ratio between the number of patients with severe ME/CFS and healthy controls who exhibit an antibody response to a peptide. The *x* axis shows the different subgroups of the antigen library. Bars above show the significant differences in Mann-Whitney-Wilcoxon two-sided tests after Bonferroni correction.

In Fig. 1F, a summary of rank sum (Mann-Whitney-Wilcoxon *U*) tests and *t* tests of ME/CFS versus healthy controls ratios is provided. See the Anti-flagellin Ig responses are overrepresented in patients with severe ME/CFS and Methods (section “Machine learning and data analysis”) for additional details on the analyses. We chose to perform both tests as we feel that *t* tests are more frequently used and easier to interpret, whereas the Mann-Whitney-Wilcoxon does not assume normal distribution, which is an assumption that does not apply to these data. Numbers of peptides in each of the subgroups (total number of peptides, and number of peptides appearing in at least one ME/CFS and at least one healthy control) are as follows: metagenomic antigens 11275 and 629; pathogenic strains 2469 and 256; probiotic strains 1609 and 138; antibody-coated strains 1999 and 133; flagellins 382 and 64; IEDB/controls 1110 and 349; and complete library 20694 and 1953.

In Fig. 3, in addition to the complete PhIP-Seq antigen library, subsets of antigens were also tested. The best-performing prevalence cutoff per subgroup is shown in the figure, while full results are provided in table S2. Additional predictions with XGB yielded lower discriminatory power and are shown in fig. S3 and table S2.

In Fig. 3 (C and D), the following abbreviations were used: #, Uniref-derived annotation (see the “Content of the PhIP-Seq microbiota antigen library, immunoprecipitation, and sequencing” section for details); ~, annotation extended by BLAST search; PSS, por secretion system; SDC, sorting domain containing; ADCP, adhesin domain containing protein.

In Fig. 4, in addition to the complete PhIP-Seq antigen library, subsets of antigens were also tested (Fig. 4D). The best-performing prevalence cutoff per subgroup is shown in Fig. 4D, while full results are provided in table S5. Additional predictions with GBR yielded lower discriminatory power and are also shown in fig. S4.

## Supplementary Materials

### Other Supplementary Material for this manuscript includes the following:

Tables S1 to S3, S5 and S6

Supporting Data and Code

## Notes

View/request a protocol for this paper from *Bio-protocol*.
