# Supplementary material for: Systemic antibody responses against human microbiota flagellins are overrepresented in chronic fatigue syndrome patients
Source: Sci Adv. 2022 Sep 23;8(38):eabq2422. doi: 10.1126/sciadv.abq2422 (PMC11580831; doi:10.1126/sciadv.abq2422)
Supplement: Supplementary file 1 — Figs. S1 to S4 Table S4 [file sciadv.abq2422_sm.pdf]

Science Advances
AAAS

Supplementary Materials for

Systemic antibody responses against human microbiota flagellins are overrepresented in chronic fatigue syndrome patients

Thomas Vogl *et al.*

Corresponding author: Thomas Vogl, thomas.vogl@aon.at; Eran Segal, eran.segal@weizmann.ac.il

Sci. Adv. **8**, eabq2422 (2022)
DOI: 10.1126/sciadv.abq2422

The PDF file includes:

Figs. S1 to S4
Table S4
Legends for tables S1 to S3, S5 and S6
Legends for supporting data and code

Other Supplementary Material for this manuscript includes the following:

Tables S1 to S3, S5 and S6
Supporting Data and Code

Supporting information

Supporting figures

Fig. S 1

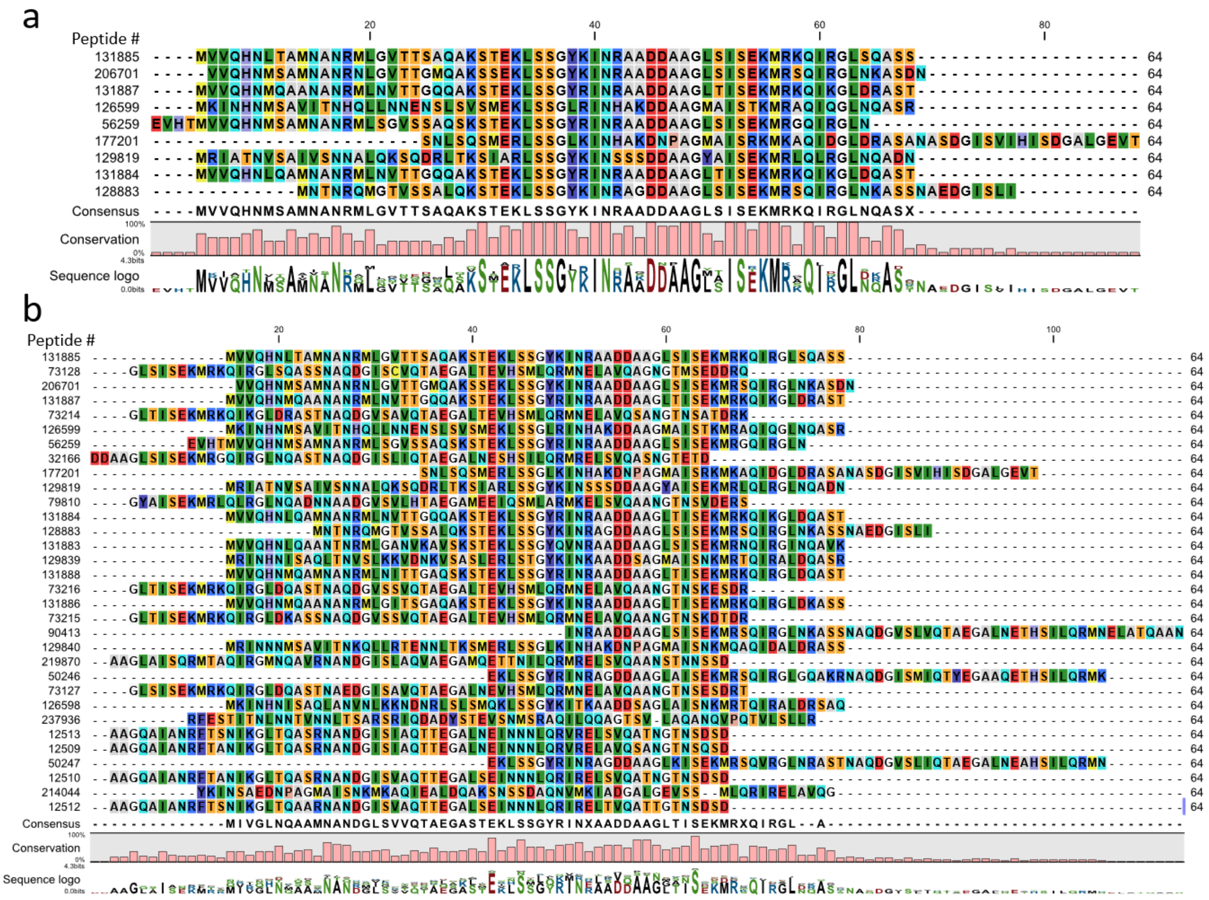

Fig. S 1: Full alignments for excerpts shown in Fig. 2b of flagellin peptides bound in ≥50% (panel a) and ≥25% (panel b) of CFS patients. The alignments of the respective peptides marked with their number (see Table S 1 for details) were generated with MegaX ((81), MUSCLE algorithm in standard settings) and visualized with CLC Main Workbench 6.

Fig. S 2

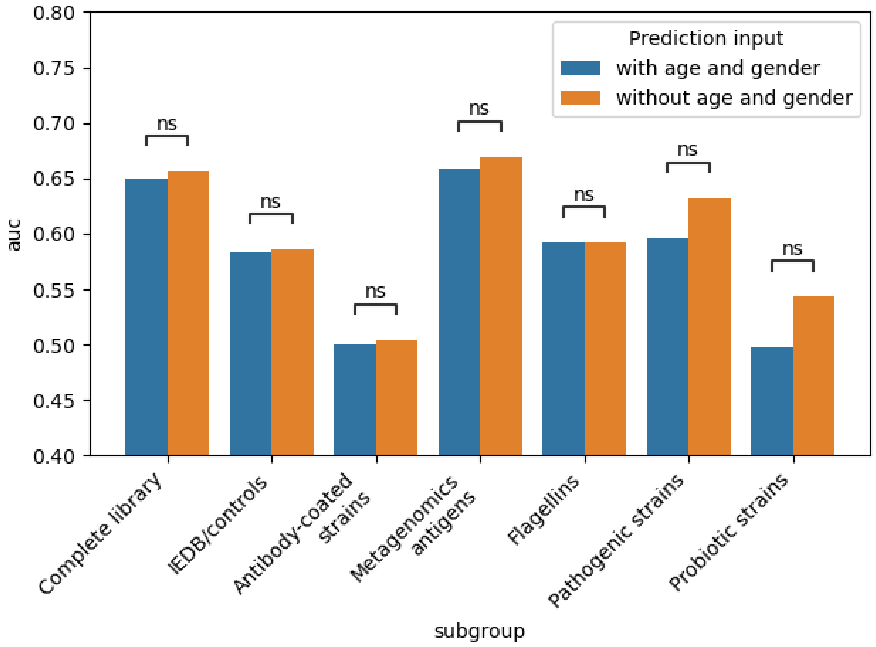

Fig. S 2: Including age and sex in the machine learning predictions does not bias classifications of ME/CFS patients from healthy controls (Fig. 3). As antibody epitope repertoires are affected by age and sex (*47*), we wanted to rule out that these factors have any influence on potential ME/CFS diagnosis from Ig epitope repertoires (Fig. 3). Therefore, we performed predictions including age/sex as features in addition to Ig responses. Predictions including age/sex information performed the same or worse than excluding them. This result can occur with GBR, if additional features without predictive value are added. Increasing noise deteriorates the model's outcome. Hence, we can conclude that age/sex effects are not biasing the classification of ME/CFS patients from healthy controls. See Table S 2 for a summary of the full predictions.

Fig. S 3

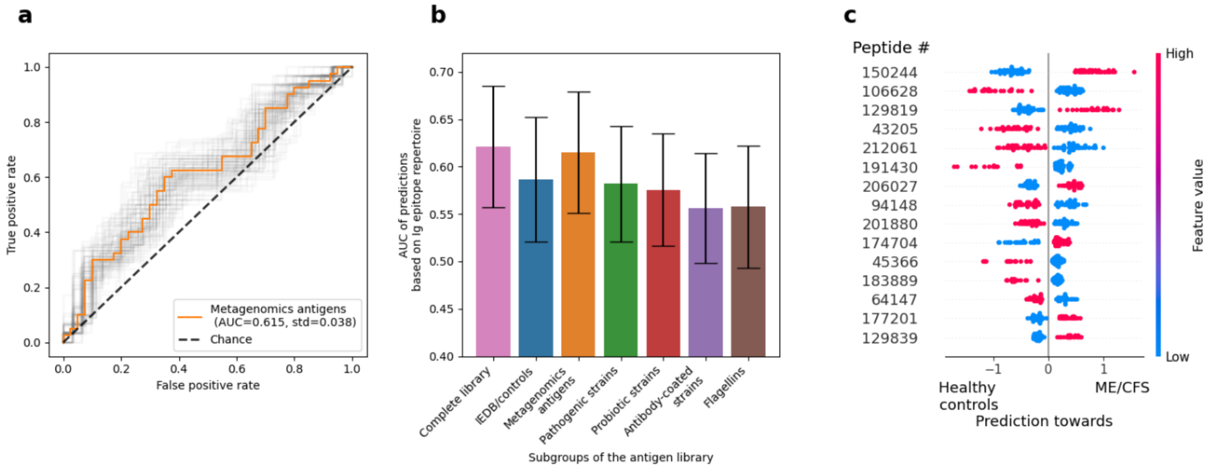

Fig. S 3: Use of an alternative algorithm (XGB (*61*)) did not improve the classification of ME/CFS patients from healthy controls based on Ig epitope repertoire data (Fig. 3). XGB differs from GBR by performing row and column subsampling. It is possible that XGB performed on this data worse than GBR, likely owing to said subsampling or due to some of the normalization performed in XGB's implementation, that might cause shifts in the data and reduce the signal.

Also full data on all cutoffs of GBR and XGB data is provided as supporting .xlsx file Table S 2.

Fig. S 4

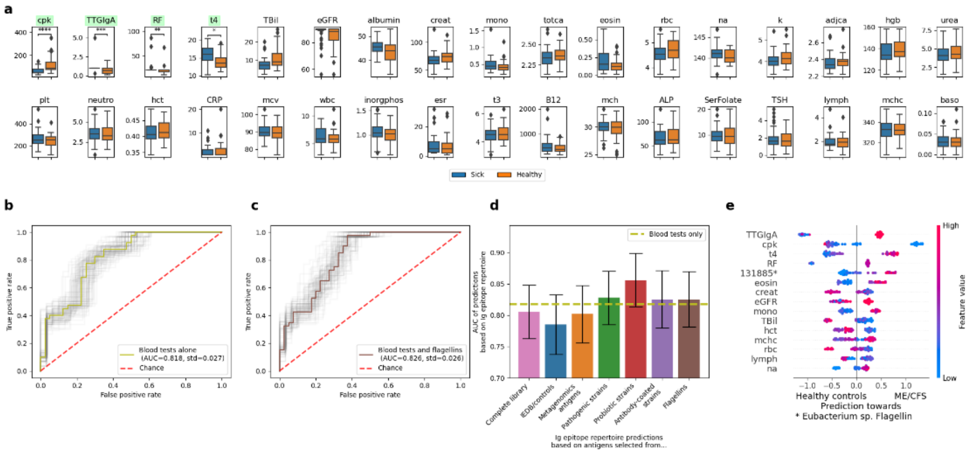

Fig. S 4: Use of the GBR algorithm (60) performed worse than XGB (61) (Fig. 4) and did not improve the classification of ME/CFS patients from healthy controls when based on combined data of blood tests and Ig epitope repertoire data (Fig. 4). GBR performed better for antibody data alone (Fig. 3 vs. Fig. S 3). XGB is a more advanced version of GBR, because it allows dealing with missing values without the need to perform imputation. As done in Fig. 3 with GBR, we also did Fig. 4 with GBR, differences are likely caused by the quality of imputation. Also, full data on all cutoffs of GBR and XGB data is provided as supporting .xlsx file (Table S 5). We have also tried to classify with linear regression, and two types of neural networks (pytorch and keran), which also did not improve beyond XGB. The classification task at hand (discriminating CFS patients from healthy controls) is challenging for machine learning algorithms for two reasons: On the one hand there is a very large number of features (=antibody binding signals against many peptides), while only a few of those features have actual power to separate CFS patients from healthy controls. So, the algorithms need to weigh the importance of these features and sort out irrelevant binding events (which is for this data set exceptionally challenging, as only a fraction of the microbiota antigens and even of the flagellins are differentially bound). On the other hand, the moderate cohort size (40 per group) alongside non-linear relationships between the features further complicates this task. Hence, many standard algorithms such as Linear Regression, even in combination with Gradient Descent, are unlikely to perform well on this data. Neural networks, that are in general very powerful at classification tasks, are typically trained on much larger datasets (and require substantially more resources). XGB/GBR are widely regarded as the most suitable algorithms for the special task required in our case of prioritizing features and handling moderate sample sizes well (while requiring relatively moderate computational resources).

Supporting tables

Table S 1

Table S 1: Supporting .xlsx file with a list of peptides bound by antibodies compared between CFS patients and healthy controls (sheet 1) as well as all flagella or flagella associated proteins detected (sheet 2).

Sheet 1: Antibody responses against peptides of bacterial and viral proteins that are bound in more than 20% of CFS patients or healthy controls and show a ≥2-fold difference in the ratio of prevalence between the groups.

Sheet 2: Any peptides of flagella-associated proteins appearing in >1 individual and the prevalence of antibody responses against them in the two groups are listed. Multiple peptides originating from the same protein are summarized. The majority of flagellins from microbiota had been annotated as such when we had initially compiled this antigen library (*47*). However, a few additional flagellins and flagella-associated proteins were missed (for example, if they had been deposited in databases such as the IEDB (*51*) or the VFDB (*52*)). These proteins were retrospectively marked as flagellins/flagella associated proteins, which is denoted in the 'Comment' column. While these proteins are listed here for the sake of completeness, they were not considered in other analyses such as predictions on subgroups of the library (as we had not manually curated the other subgroups).

Table S 2

Table S 2: Full data on all cutoffs of GBR and XGB predictions based on antibody repertoire data is provided as supporting .xlsx file. Sheets: "predictions_summary_GBR" – Summary of data shown in Fig. 3, "predictions_summary_xgboost" – Summary of data shown in Fig. S 3, "predictions_summary_GBR_age+sex" Summary of data shown in Fig. S 2.

Table S 3

Table S 3: Supporting .xlsx file peptides identified by SHAP analysis to drive GBR predictions of ME/CFS patients from healthy controls from antibody epitope repertoires (Fig. 3).

Table S 4

Table S 4: Details on conventional blood tests data available for the cohort (obtained from the UKMEB (58, 63)) including abbreviations used in Fig. 4. Details on the exact hematological and biochemical parameters assessed (as well as ranges) are provided in. Blood tests excluded due to an imbalance in the missingness between the two groups are marked.

Abbreviation	Details	Description	Comment
wbc	Full blood count (FBC) - WBC. 10^9/L. Number to 2 decimal places	range: (2.6,16.9)	
plt	Full blood count (FBC) - Platelets (PLT). 10^9/L. Number (up to 3 digits)	range: (115,531)	
rbc	Full blood count (FBC) - RBC. 10^12/L. Number to 2 decimal places	range: (3.69,7.03)	
hgb	Full blood count (FBC) - Haemoglobin. g/L. Number (up to 3 digits)	range: (101,184)	
hct	Full blood count (FBC) - Haematocrit. Number to 3 decimal places	range: (.313,.538)	
mcv	Full blood count (FBC) - Mean corpuscular volume (MCV). fl. Number to 1 decimal	range: (68,104)	
mch	Full blood count (FBC) - Mean corpuscular haemoglobin (MCH). pg. Number to 1 dec	range: (20.9,34.9)	
mchc	Full blood count (FBC) - Mean corpuscular hemoglobin concentration (MCHC). g/L.	range: (32,374)	
neutro	Full blood count (FBC) - Neutrophils. 10^9/L. Number to 2 decimal places	range: (.98,12.8)	
lymph	Full blood count (FBC) - Lymphocytes. 10^9/L. Number to 2 decimal places	range: (.32,9.65)	
mono	Full blood count (FBC) - Monocytes. 10^9/L. Number to 2 decimal places	range: (.12,1.55)	
eosin	Full blood count (FBC) - Eosinophils. 10^9/L. Number to 2 decimal places	range: (0,.8)	
baso	Full blood count (FBC) - Basophils. 10^9/L. Number to 2 decimal places	range: (0,.13)	
na	Urea & Electrolytes - Sodium. mmol/L. Number (up to 3 digits)	range: (130,147)	
k	Urea & Electrolytes - Potassium. mmol/L. Number to 1 decimal place	range: (3.2,5.9)	
urea	Urea & Electrolytes - Urea. mmol/L. Number to 1 decimal place	range: (1.5,14)	
creat	Urea & Electrolytes - Creatinine. umol/L. Number (up to 3 digits)	range: (42,240)	
totca	Urea & Electrolytes - Total calcium. mmol/L. Number to 2 decimal places	range: (2.02,2.76)	
adjca	Urea & Electrolytes - Adjusted calcium. mmol/L. Number to 2 decimal places	rnage: (2.15,2.76)	
inorgphos	Urea & Electrolytes - Inorganic phosphate. mmol/L. Number to 2 decimal places	range: (.4,2.33)	
eGFR	Urea & Electrolytes - Estimated glomerular filtration rate (eGFR)	n.a.	
TBil	Liver Function Tests - Total bilirubin. umol/L. Number (up to 2 digits)	n.a.	
albumin	Liver Function Tests - Albumin. g/L. Number (up to 2 digits)	range: (29,54)	
ALP	Liver Function Tests - Alkaline Phosphatase (ALP). U/L. Number (up to 3 digits)	n.a.	
ALT	Liver Function Tests - Alanine aminotransferase (ALT/STGO). U/L. Number (up to 3 digits)	n.a.	
ast	Liver Function Tests - Aspartate aminotransferase (AST/SGP). U/L. Number (up to 3 digits)	range: (10,96)	*
cpk	Creatine phosphokinase (CPK) - Creatine phosphokinase (CPK). U/L. Number (up to 3 digits)	range: (12,933)	
t3	Thyroid function - Free T3. pmol/L. Number to 1 decimal place	range: (2.7,7.3)	
t4	Thyroid function - Free T4. pmol/L. Number to 1 decimal place	range: (9,34.9)	
TSH	Thyroid function - TSH. mU/L. Number to 2 decimal places	n.a.	
SerFolate	Folate - Serum folate. ng/mL. Number to 1 decimal place	n.a.	
esr	Rheumatological tests - Erythrocyte sedimentation rate. mm/h. Number (up to 3 digits)	range: (1,57)	
CRP	Rheumatological tests - C Reactive protein (CRP)	n.a.	
RF	Rheumatological tests - Rheumatoid factor	n.a.	
TTGIgA	Coeliac Screen - Tissue transglutaminase antibody - IgA	n.a.	
B12	Coeliac Screen - Tissue transglutaminase antibodies - IgG	n.a.	

*Excluded in Fig. 4 due to lack of full data (see Methods section)

Table S 5

Table S 5: Full data on all cutoffs of XGB predictions based on the combination of antibody repertoire data and blood tests is provided as supporting .xlsx file. “predictions_summary_GBR” – Summary of data shown in Fig. 4, “predictions_summary_xgboost” – Summary of data shown in Fig. S 4.

Table S 6

Table S 6: Supporting .xlsx file peptides identified by SHAP analysis to drive GBR predictions of ME/CFS patients from healthy controls from combined data of blood tests and antibody epitope repertoires (Fig. 4).
