# Supplementary figures and images for: Systemic antibody responses against human microbiota flagellins are overrepresented in chronic fatigue syndrome patients
Source: Sci Adv. 2022 Sep 23;8(38):eabq2422. doi: 10.1126/sciadv.abq2422 (PMC11580831; doi:10.1126/sciadv.abq2422)

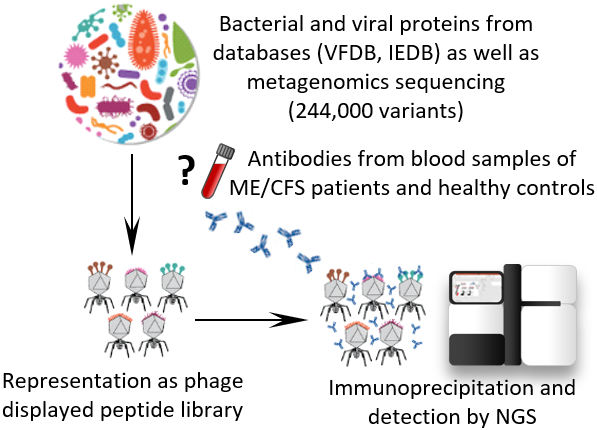

Supplement: Supplementary file 3 — Supporting Data and Code [file sciadv.abq2422_supporting_data_and_code.zip › PhageIPSeq_CFS-main/PhageIPSeq_CFS/Figures/figure_1/Fig.1a.png]

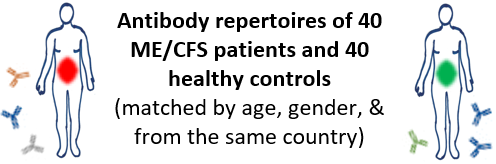

Supplement: Supplementary file 3 — Supporting Data and Code [file sciadv.abq2422_supporting_data_and_code.zip › PhageIPSeq_CFS-main/PhageIPSeq_CFS/Figures/figure_1/Fig.1b.png]

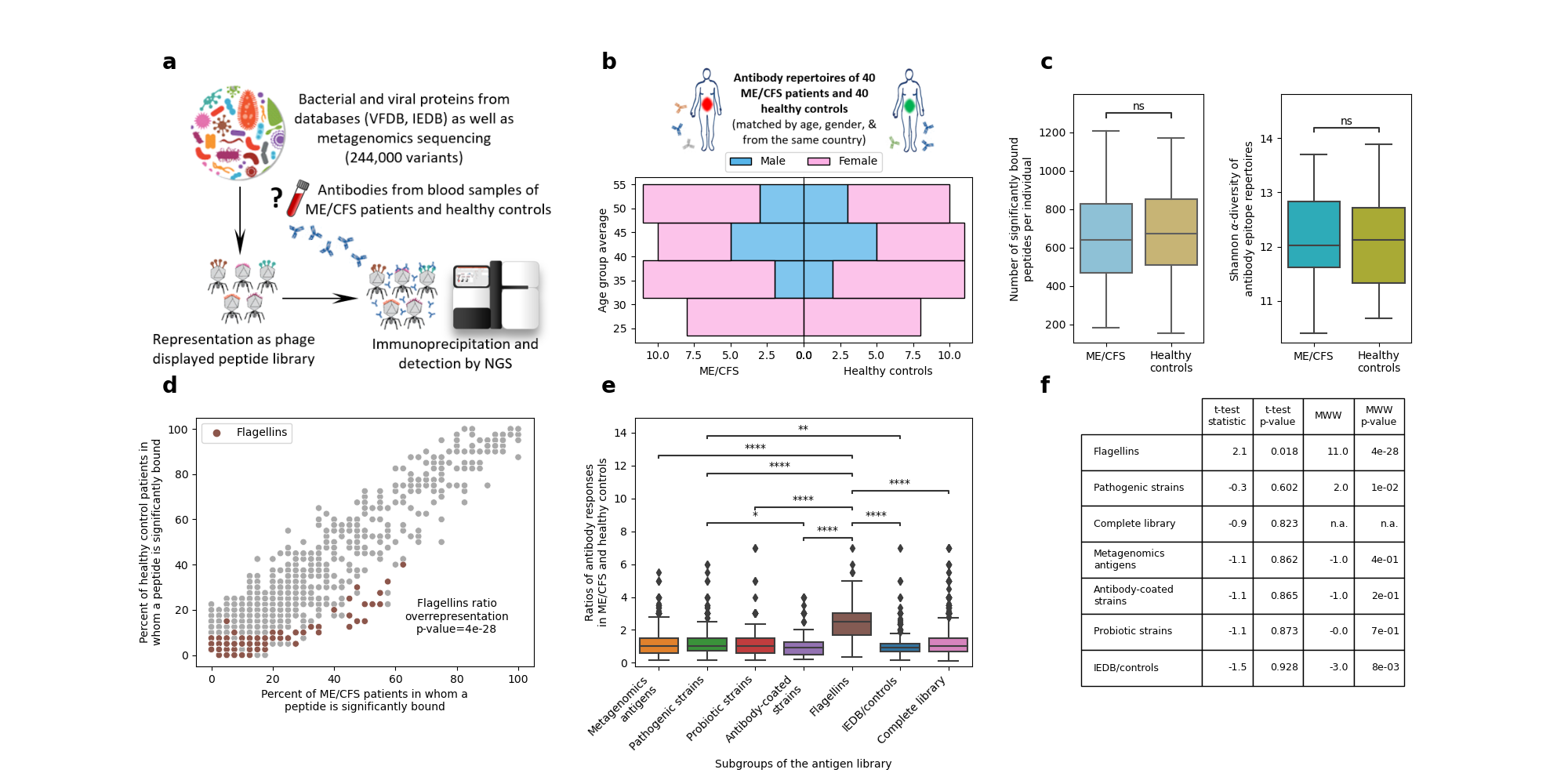

Supplement: Supplementary file 3 — Supporting Data and Code [file sciadv.abq2422_supporting_data_and_code.zip › PhageIPSeq_CFS-main/PhageIPSeq_CFS/Figures/figure_1/figure_1.png]

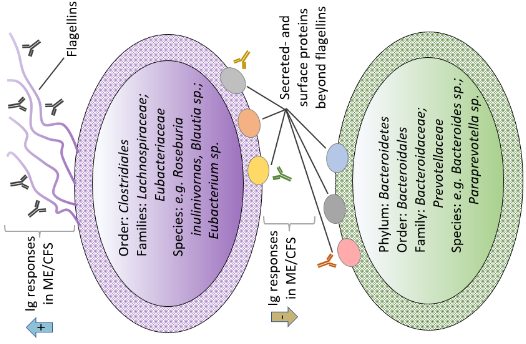

Supplement: Supplementary file 3 — Supporting Data and Code [file sciadv.abq2422_supporting_data_and_code.zip › PhageIPSeq_CFS-main/PhageIPSeq_CFS/Figures/figure_3/Fig.3e.png]

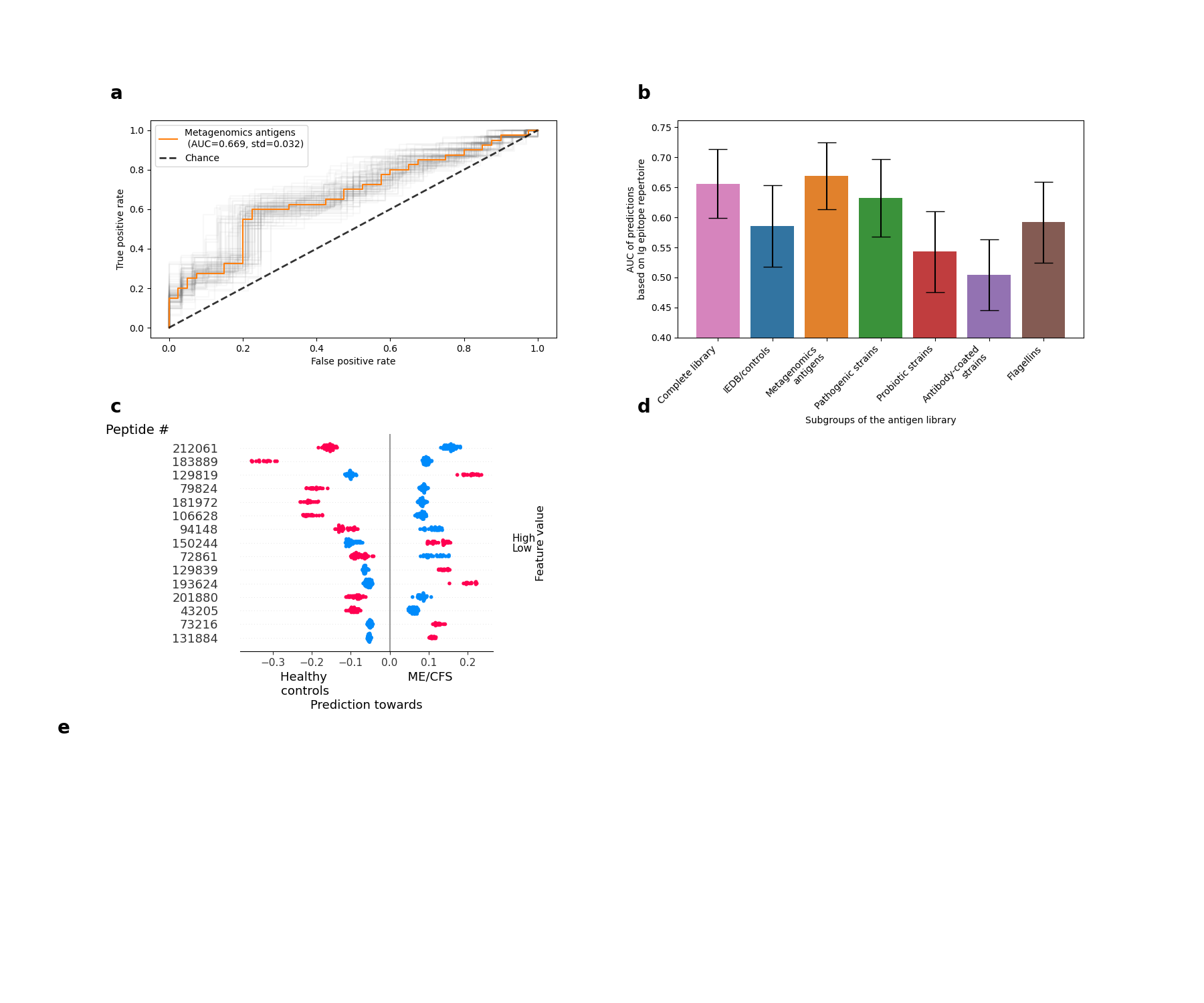

Supplement: Supplementary file 3 — Supporting Data and Code [file sciadv.abq2422_supporting_data_and_code.zip › PhageIPSeq_CFS-main/PhageIPSeq_CFS/Figures/figure_3/figure_3_GBR.png]

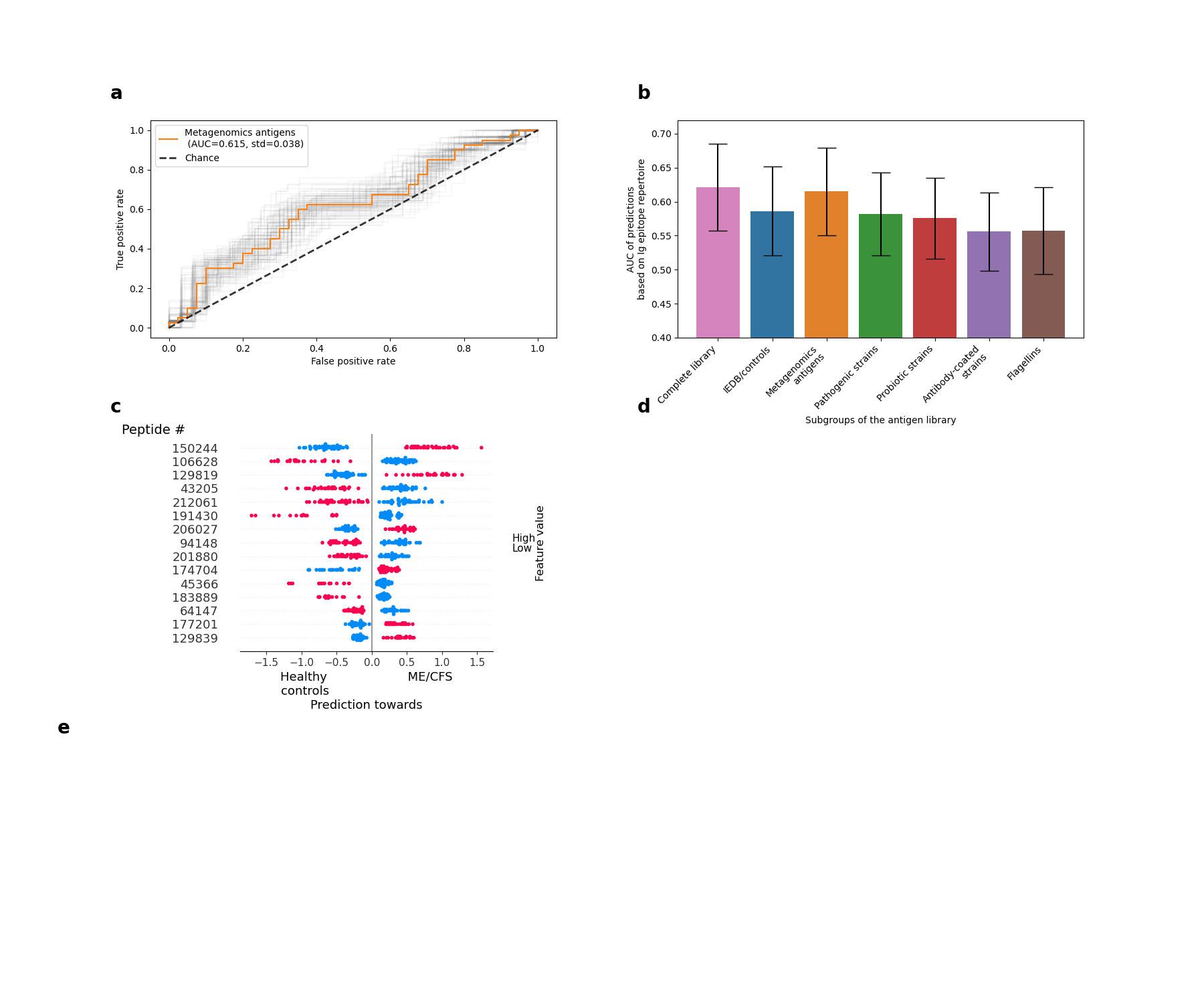

Supplement: Supplementary file 3 — Supporting Data and Code [file sciadv.abq2422_supporting_data_and_code.zip › PhageIPSeq_CFS-main/PhageIPSeq_CFS/Figures/figure_3/figure_3_xgboost.png]

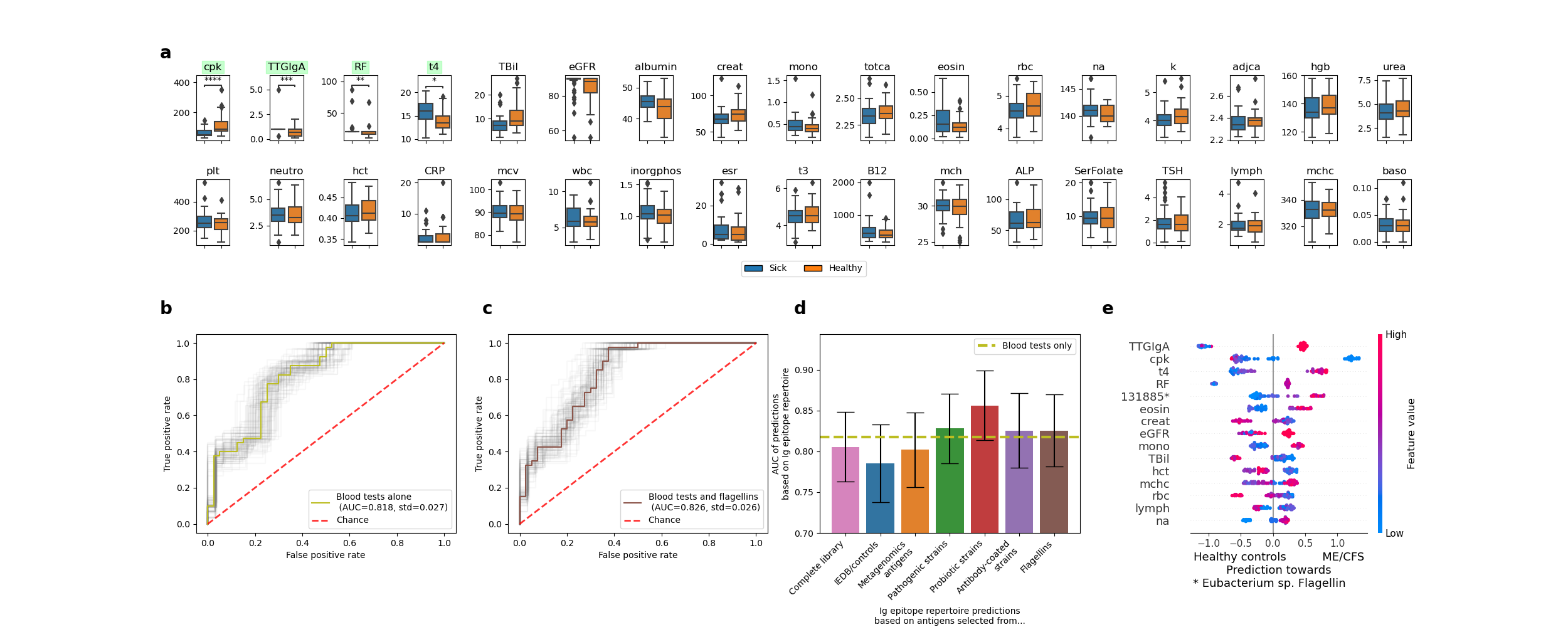

Supplement: Supplementary file 3 — Supporting Data and Code [file sciadv.abq2422_supporting_data_and_code.zip › PhageIPSeq_CFS-main/PhageIPSeq_CFS/Figures/figure_4/figure_4_GBR.png]

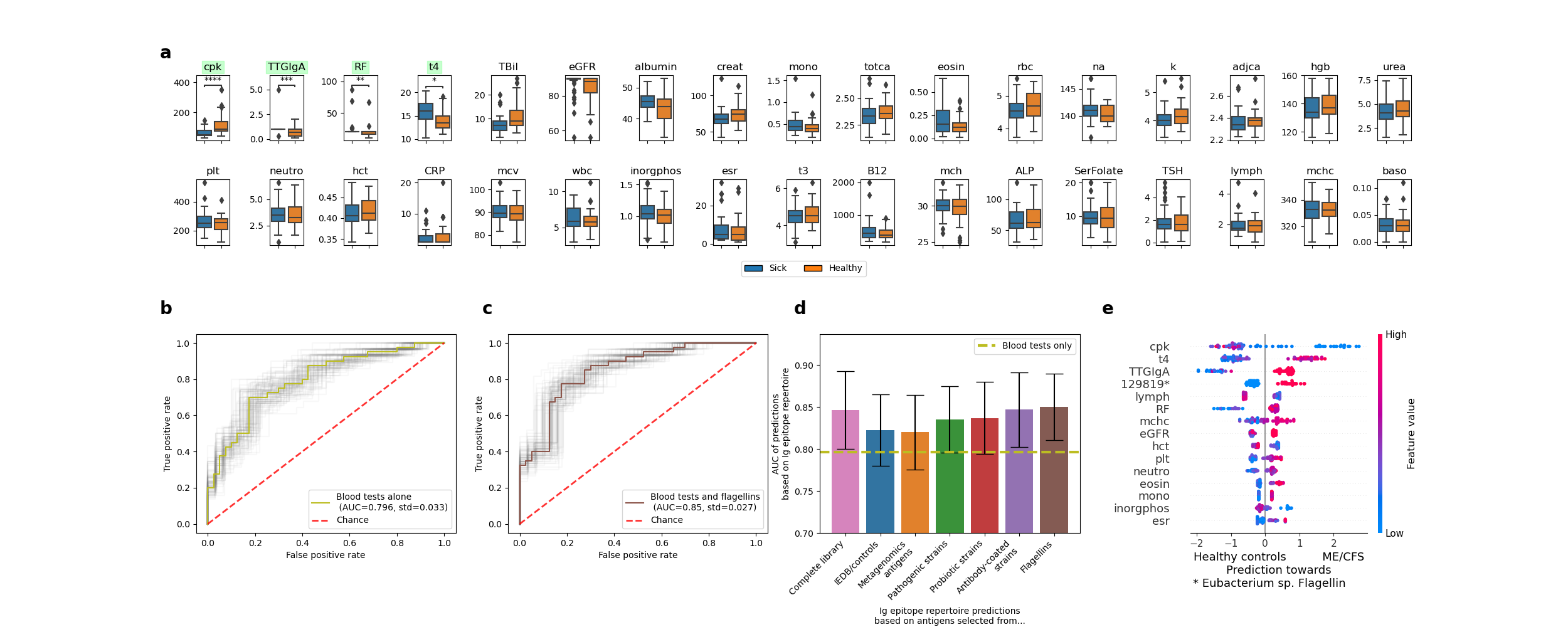

Supplement: Supplementary file 3 — Supporting Data and Code [file sciadv.abq2422_supporting_data_and_code.zip › PhageIPSeq_CFS-main/PhageIPSeq_CFS/Figures/figure_4/figure_4_xgboost.png]

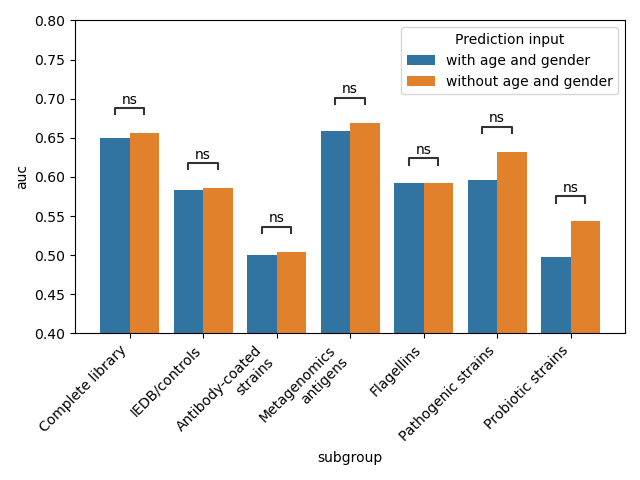

Supplement: Supplementary file 3 — Supporting Data and Code [file sciadv.abq2422_supporting_data_and_code.zip › PhageIPSeq_CFS-main/PhageIPSeq_CFS/Figures/supp_figures/supp_fig_7_GBR.png]

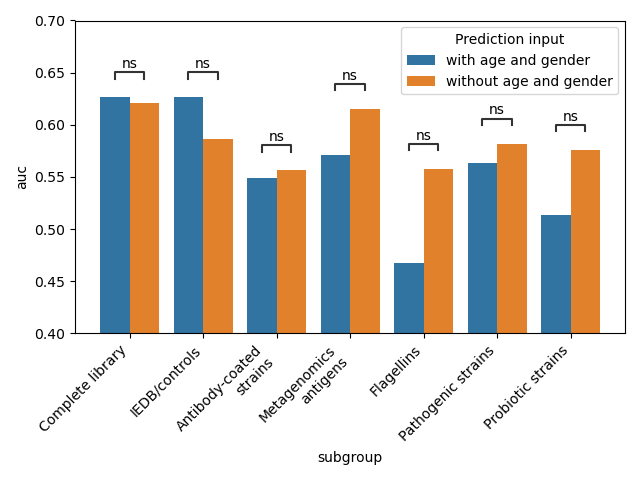

Supplement: Supplementary file 3 — Supporting Data and Code [file sciadv.abq2422_supporting_data_and_code.zip › PhageIPSeq_CFS-main/PhageIPSeq_CFS/Figures/supp_figures/supp_fig_7_xgboost.png]

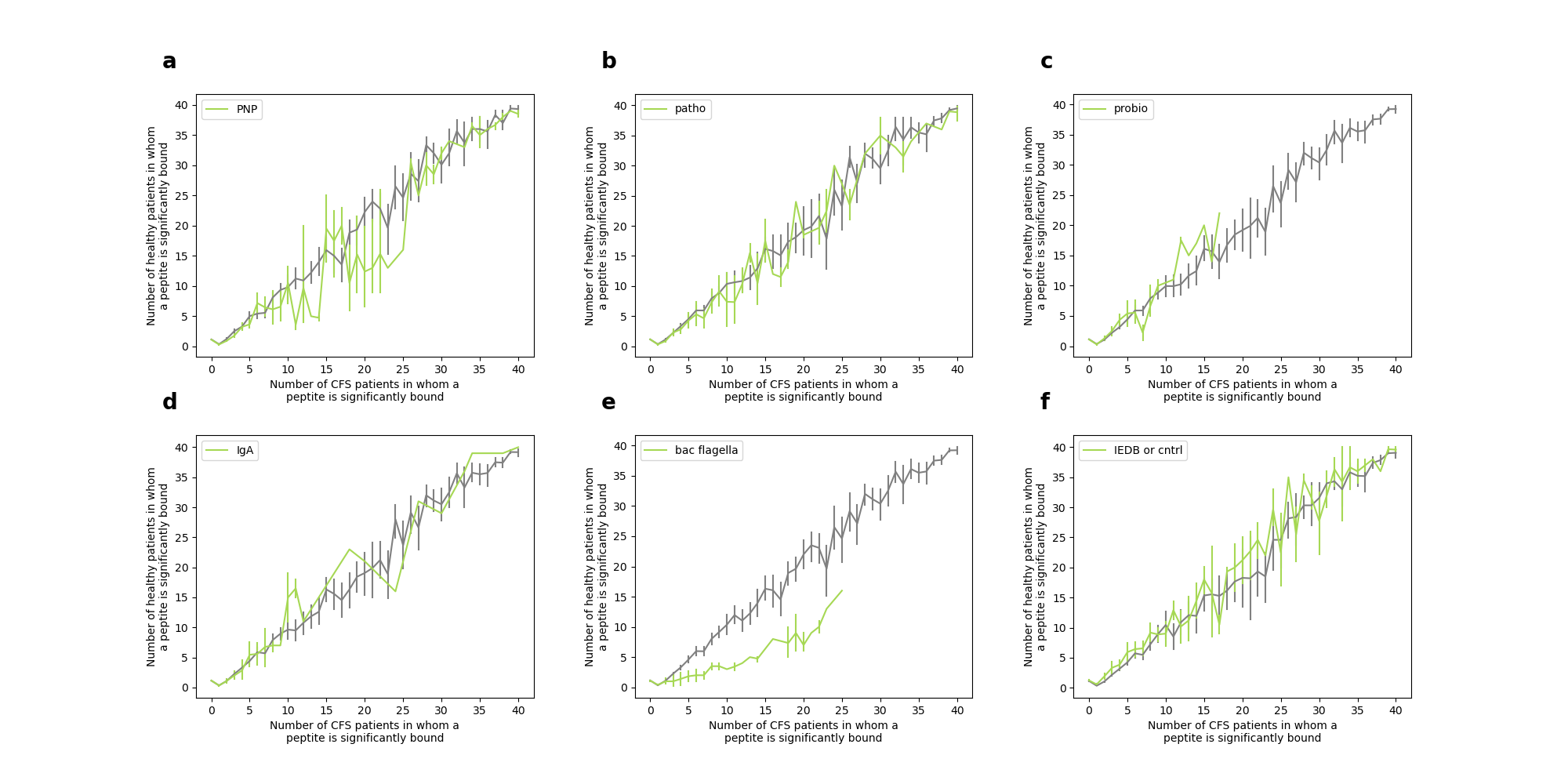

Supplement: Supplementary file 3 — Supporting Data and Code [file sciadv.abq2422_supporting_data_and_code.zip › PhageIPSeq_CFS-main/PhageIPSeq_CFS/Figures/supp_figures/supp_figure_1.png]
